# Cobalt mitigates zinc-starvation effects in *Pseudomonas aeruginosa*

**DOI:** 10.1007/s10534-025-00769-4

**Published:** 2025-11-27

**Authors:** Emma Michetti, Valerio Secli, Maria Luisa Astolfi, Chiara Demingo, Francesca Pacello, Serena Ammendola, Andrea Battistoni

**Affiliations:** 1 Department of Biology, Tor Vergata University of Rome, Via Della Ricerca Scientifica, 00133 Rome, Italy; 2 Department of Chemistry, Sapienza University of Rome, P.Le Aldo Moro 5, 00185 Rome, Italy; 3 PhD in Cellular and Molecular Biology, Tor Vergata University of Rome, Rome, Italy

**Keywords:** Zinc starvation, Cobalt, *Pseudomonas aeruginosa*, Metalloproteins, Metal homeostasis, Zur

## Abstract

Bacterial pathogens must rapidly adapt to fluctuating metal availability within the host, where essential micronutrients are actively sequestered as part of nutritional immunity. Among these, zinc is a critical cofactor for a wide array of enzymes and regulatory proteins, and its availability is tightly linked to the expression of key virulence traits in *Pseudomonas aeruginosa*. This opportunistic pathogen employs different zinc acquisition systems transcriptionally regulated by the Zinc Uptake Regulator Zur, enabling its persistence within the host. Recently, Zur-controlled operons involved in the uptake/export of cobalt have been identified. Although cobalt is primarily associated with cobalamin-dependent reactions, its selective import under zinc-limiting conditions suggests a potential role for cobalt in bacterial adaptation to zinc scarcity. Yet, the functional relevance of this metal-based compensation remains poorly defined. This study shows that cobalt supplementation alleviates key effects of severe zinc deficiency in *P. aeruginosa*, including reduced pyocyanin production, impaired swarming motility, and enhanced sensitivity to oxidative stress. Furthermore, in vitro assays demonstrate that cobalt can functionally replace zinc in the proteases LasA and LasB and the transcriptional regulator Zur. Finally, we found that a *P. aeruginosa* strain deficient in the pyochelin-cobalt receptor PA2911 exhibits impaired colonization of *Galleria mellonella* larvae, supporting the hypothesis that cobalt compensatory function may be crucial during infection. Our results suggest that cobalt may play a broader biological role than previously recognized, highlighting its potential to support *P. aeruginosa* survival and pathogenicity in zinc-limiting environments.

**Graphical abstract:**

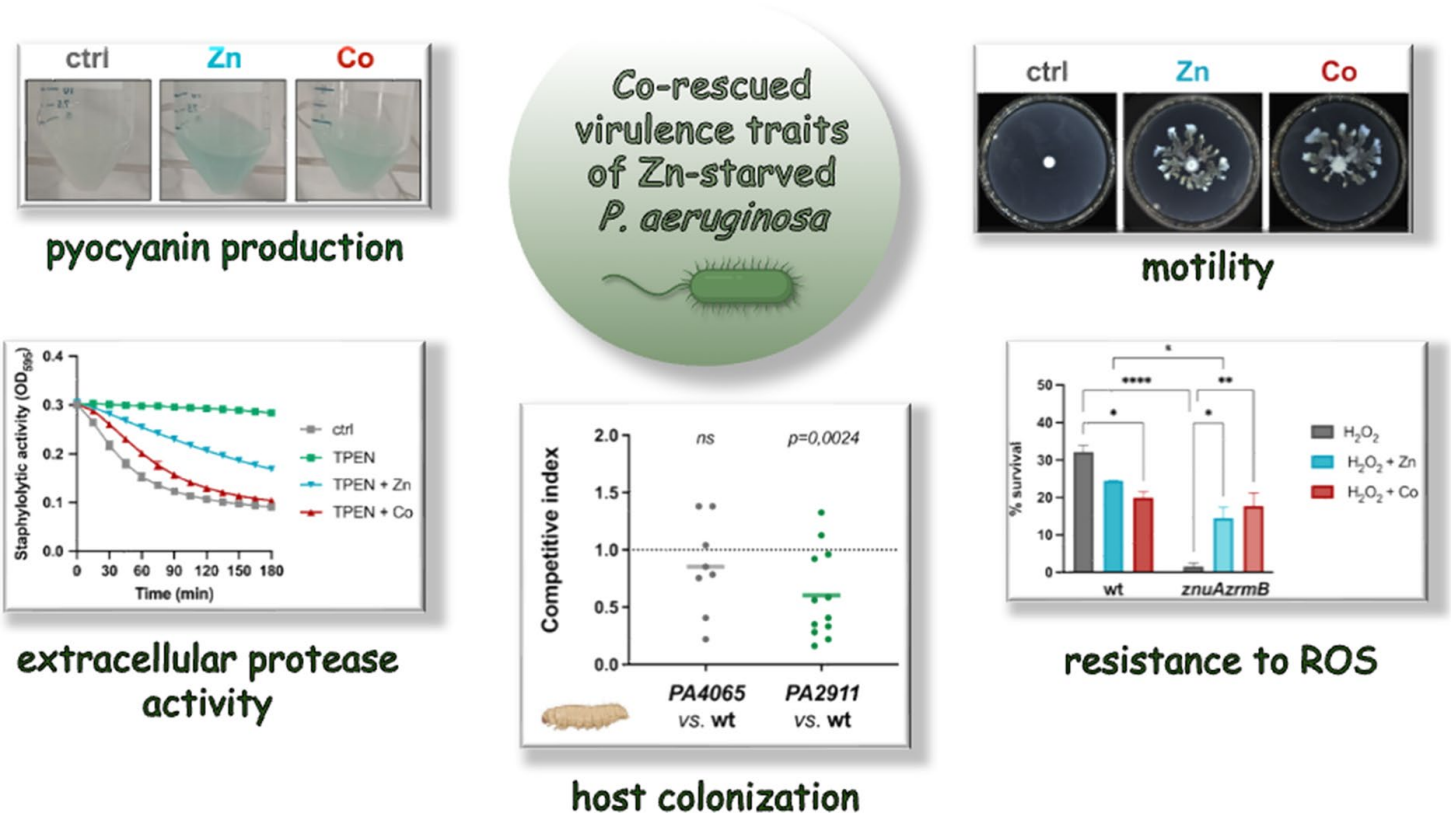

**Supplementary Information:**

The online version contains supplementary material available at 10.1007/s10534-025-00769-4.

## Introduction

Trace metals, including iron (Fe), zinc (Zn), copper (Cu), cobalt (Co), manganese (Mn), and nickel (Ni), are micronutrients required for fundamental biological functions across all domains of life (Maret 2010). Among these, Fe, Zn, Cu, and Mn have broad functional roles as enzymatic cofactors in key pathways, such as DNA replication and repair, cell division, and cellular respiration. In contrast, Ni and Co participate in more specialized but still essential processes (Alfano and Cavazza 2020).

Given the essentialness of trace metals, their bioavailability must be ensured, but their intracellular concentration must be tightly regulated, since excess can cause toxicity either by promoting oxidative damage or by inducing mis-metalation, an improper incorporation of metal ions into proteins, affecting their folding, structure and function (Chandrangsu et al. 2017). To avoid mis-metallation, bacteria have evolved strategies to fine-tune intracellular metal content, including uptake/efflux systems, metallochaperones, and transcriptional regulators (Waldron et al. 2009; Imlay 2014; Barwinska-Sendra and Waldron 2017).

Interestingly, many proteins can accommodate non-native metals, allowing cells to maintain enzyme function under conditions of metal scarcity. For instance, cambialistic superoxide dismutases can incorporate either Mn or Fe, depending on availability, retaining activity under varying metal conditions (Meier et al 1982; Gabbianelli et al 1995). This flexibility may contribute to the ability of pathogens to overcome metal starvation imposed by host metal sequestration strategies (Garcia et al. 2017). Moreover, several studies have revealed that the possibility of replacing Fe with Mn in several mononuclear enzymes makes these proteins less susceptible to the Fenton reaction-induced damage, increasing the resistance of cells to oxidative stress conditions (Imlay 2014; Rohaun et al. 2024).

Even if most known examples come from the crosstalk between Fe and Mn, recent evidence suggests a role for Co in mitigating specific metal imbalances. Co primarily serves as the cofactor of cobalamin, or vitamin B12, but is also found in a few mononuclear enzymes such as methionine aminopeptidase, prolidase, and nitrile hydratase (Okamoto and Eltis 2011). Co is typically regarded as an essential metal with limited in vivo roles and potential high toxicity, mainly due to mis-metallation and consequent inactivation of [Fe-S] enzymes under conditions of excess Co exposure (Ranquet et al. 2007). However, *Salmonella* Typhimurium can use Co to restore virulence-related phenotypes impaired by Zn starvation, likely by substituting the native Zn with Co in some metalloproteins, and it was recently proposed that *Pseudomonas aeruginosa* has a pyochelin-Co (PCH-Co) uptake system which responds specifically to Zn starvation (Ammendola et al. 2020; Secli et al. 2024).

The ability to overcome Zn starvation is crucial for full bacterial virulence. Thus, limiting Zn availability is a nutritional immunity strategy conserved across organisms towards different pathogens, including *P. aeruginosa* (Murdoch and Skaar 2022; Secli et al. 2023; Michetti et al. 2025). This pathogen is a leading cause of mortality in patients affected by cystic fibrosis (CF), and its exceptional ability to efficiently import Zn is pivotal in evading the host nutritional immunity response and establishing pulmonary infections (Malhotra et al. 2019). Zn intracellular concentration is sensed by the Zinc Uptake Regulator Zur, which in *P. aeruginosa* regulates a broad set of genes, including many with still-unknown functions (Pederick et al. 2015). In this microorganism, the Zur-regulated response to Zn starvation includes the induction of high-affinity Zn importers such as the ZnuABC transporter and the ZrmABCD, also known as CntOLMI, system (Mastropasqua et al. 2017; Lhospice et al. 2017). Zur also controls the expression of Zn-independent paralogs of different proteins, including the ribosomal proteins L31 and L36, the virulence-associated transcription factors DksA2, and the GTP cyclohydrolase FolE. Additionally, Zur regulates several genes encoding putative metal transporters, metal chaperones, and enzymes, many of which remain functionally uncharacterized (Gabriel and Helmann 2009; Furman et al. 2013; Mastropasqua et al. 2017; Ducret et al. 2022).

In a recent work, we demonstrated that among the *P. aeruginosa* operons controlled by Zur, two of them are involved in PCH-Co trafficking, and we suggested that the controlled import of Co may serve as an adaptive strategy to survive in Zn-depleted environments (Secli et al. 2024). However, the specific contribution of Co in the *P. aeruginosa* adaptation to Zn-starvation remains poorly understood and has been explored only to a limited extent. The present study reveals that Co can restore, in vitro, some virulence phenotypes impaired under Zn-starved conditions in *P. aeruginosa*, and that the disruption of the PCH-Co uptake system decreases the fitness of *P. aeruginosa* in a *G. mellonella* infection model. Additionally, we demonstrate that, like Zn, Co modulates the transcription of the Zur-regulon and triggers Zur binding to its target promoters. These findings suggest that Co may play a compensatory role in the Zn-starvation response, with potential implications for *P. aeruginosa* virulence.

## Materials and methods

### Bacterial strains and growth conditions

Bacterial strains (listed in Table 1) were streaked from glycerol stocks on *Pseudomonas* Isolation Agar (PIA; Merck) or Luria–Bertani (LB: Tryptone 10 g L^−1^, yeast extract 5 g L^−1^, NaCl 10 g L^−1^) agar, supplemented with antibiotic when needed (for *Escherichia coli*, gentamicin 10 mg L^−1^, ampicillin 100 mg L^−1^, kanamycin 50 mg L^−1^ and carbenicillin 100 mg L^−1^; for *P. aeruginosa* gentamicin 100 mg L^−1^ and carbenicillin 300–500 mg L^−1^) and incubated at 37 °C. Liquid cultures were routinely grown in LB at 37 °C under shaking. *Staphylococcus aureus* SH1000 was grown at 37 °C in Brain Heart Infusion (BHI, Becton Dickinson) broth. Vogel-Bonner Minimal Medium (MgSO_4_-7H_2_O 0,192 g L^−1^, citric acid 2 g L^−1^, anhydrous K_2_HPO_4_ 10 g L^−1^, NaNH_4_HPO_4_—4H_2_O 3.5 g L^−1^, glucose 2 g L^−1^, FeSO_4_ 2 μM) supplemented with the chelating agent EDTA 5 µM (E-VBMM) was used for bacterial growth under Zn limiting conditions, as already described (Mastropasqua et al. 2017). To minimize Zn contaminations, VBMM was prepared in disposable plastic containers and sterilized by filtration in Vacuum Filter-Storage Bottle Systems, 0.22 μm (Corning). Additionally, Zn-limiting conditions were also achieved using freshly prepared Artificial Sputum Medium (ASM: 5 g L^−1^ mucin from pig stomach mucosa, 4 g L^−1^ low molecular-weight salmon sperm DNA, 5.9 mg L^−1^ diethylene triaminepentaacetic acid (DTPA), 5 g L^−1^ NaCl, 2.2 g L^−1^ KCl, 1.81 g L^−1^ Tris base, 5 g L^−1^ Casamino acids, 5 mL L^−1^ egg yolk emulsion) (Kirchner et al. 2012).

**Table 1:** Bacterial strains and plasmids

*P.aeruginosa* **PA14** **strains**	**Relevant** **genotype**	**Reference/source**
wild-type		Lab collection
*znuA*	*znuA*::Gm	D’Orazio et al. (2015)
*znuAzrmB*	*znuA*::scar *zrmB*::Gm	Secli et al. (2024)
*PA2911*	*PA2911*::Gm	This work
*PA4065*	*PA4065*::Gm	Secli et al. (2024)
Other strains	Relevant genotype	Source
*E. coli* DH5α	φ80 ∆*lac*Z 15∆ (*lac*-*arg*F) U169 *deo*R*rec*A1 *end*A1 *hsd*R17 (rk^−^, mk^+^) *pho*A*sup*E44 λ^−^*thi*-1	Lab collection
*E. coli* HB101	pRK2013	Lab collection
*Staphylococcus aureus* SH1000		Lab collection

**Plasmids**	**Description**	**Reference/source**
pASK-Zur	Strep-tagged Zur expression vector, Amp^R^	Ellison et al. (2013)
pEX18Tc	Broad-host-range gene replacement; sacB + ;Tc^R^,oriT +	Hoang et al. (1998)
P*zrmA* pETS-lux	Broad-host-range, *luxCDABE* carrying *zrmA* promoter; Gm^R^	Michetti et al. (2025)
pPS856	Source of gentamicin resistance cassette. Amp^R^, Gm^R^	Hoang et al. (1998)
pRK2013	Broad-host-range helper vector; Kan^R^	Lab collection
p*fliE*pTZ110	*fliE* promoter cloned in pTZ110	Mastropasqua et al. (2018)

### Metal analysis

Overnight LB cultures of PA14 wild-type and the *znuA* mutant strain were grown in E-VBMM containing a cocktail of transition metals in trace amounts (0.1 μM FeSO_4_, NiSO_4_, Co(NO_3_)_2_, CuSO_4_ and MnCl_2_) and increasing ZnSO_4_ concentrations (0.2, 0.4, and 0.6 μM). Cultures were grown for 18 h at 37 °C in 50 mL polypropylene tubes. After overnight growth, aliquots of 10 mL of the cell cultures were collected and centrifuged at 5000 × g for 15 min. Then, the pellet was washed with 10 mL of phosphate buffer saline (PBS) containing 1 mM EDTA to remove excess metals. Subsequently, cell pellets were freeze-dried and accurately weighed. Metal content in bacterial pellets was determined by inductively coupled plasma mass spectrometry (ICP-MS), as described previously (D’Orazio et al. 2015; Secli et al. 2024).

### Zur expression and purification

The expression and purification of *P. aeruginosa* Zur were performed following a previously described protocol with minor modifications (Ellison et al. 2013). Briefly, overnight cultures of *E. coli* DH5α carrying pASK-Zur (Table 1) were used to inoculate 5 L of LB supplemented with ampicillin. The cultures were grown at 37 °C with aeration at 180 rpm until the OD_600_ reached 0.6, at which time anhydrotetracycline was added to a final concentration of 0.3 µg mL^−1^. The cultures were then incubated at 30 °C with aeration at 120 rpm for 3 h. All subsequent steps were performed on ice or at 4 °C. Cells were harvested by centrifugation at 5000 × g for 15 min, and the pellets were suspended in 10 mL lysis buffer (10 mM Tris–HCl, pH 8.0, 40 mM KCl, 10 mM MgCl_2_, 100 mM DTT, 5 mM octyl β-D-glucopyranoside, 100 µM ZnSO_4,_ and 5% glycerol). The cells were lysed by sonication using a Branson Sonifer SLPe (30 amplitude, 10 s on, and 16 s off until complete lysis), and 5 µg mL^−1^ DNase was added for 15 min. The lysate was centrifuged at 5000 × g for 15 min to remove the insoluble fraction and was applied to the Strep-tactin gravity flow column (IBA GMH) according to the manufacturer’s instructions. Protein fractions were pooled and dialyzed in storage buffer (10 mM Tris–HCl, pH 8.0, 40 mM KCl, 10 mM MgCl_2_, 5 mM DTT, and 10% glycerol) and concentrated using a Centricon Ultra centrifugal filter (Sigma Aldrich) with a cutoff of 3500 Da. The protein concentration was determined by Bradford assays (Bio-Rad), and the protein was stored at − 20 °C.

### Electromobility shift binding assay (EMSA)

For the EMSA experiments, the DNA promoters were obtained by PCR using primers listed in Table 2, purified with the DNA clean-up and concentration (Zymo Research), and the final elution step was performed in MilliQ water. DNA promoter concentrations were determined with a NanoDrop™ Lite Spectrophotometer (Thermo Fisher Scientific). Binding assays were performed according to the procedure already described (Ducret et al. 2020). Briefly, reactions were performed with a mixture composed of the 5X Zn-less Binding Buffer (50 mM Tris, 200 mM KCl, 50 mM MgCl_2_, 5 mM DTT, and 25% Glycerol), 30 ng of DNA, with or without 1 µM Zur protein, 30 μM TPEN, and different concentrations of ZnSO_4_, CoCl_2_ or CuSO_4_ at room temperature for 30 min. Denatured Zur (Zur*) for negative protein control was obtained by heating the protein at 100 °C for 30 min. Samples were then run at 4 °C on a 7.5% polyacrylamide native gel containing 2.5% glycerol in Tris Borate Buffer. Electromobility of the bands was analyzed by staining the gel with 0.1% ethidium bromide and revealed under UV light using a ChemiDoc imaging system (Bio-Rad) and the relative abundance of bands was quantified using ImageJ.

**Table 2:** Primers

Target	Forward (5′-3′)	Reverse (5′-3′)	Source
*P. aeruginosa* promoter			
*zrmA*	GGCTGGGCTGGTCGTCGGA	CTGCGGGACTTTCCGTTTCC	This work
*pchE*	CCATCGCTTTCCGCAAGC	CTAGGGCAAGCATCTGAG	This work
*P. aeruginosa PA2911* mutant			
5’ *PA2911*	ATAGAATTCGGTCTCACCAATCCGGTT	ATAGGATCCATCGATGTGGTCGAGTT	This work
3’ *PA2911*	TATGGATCCGACGAAACGATCGACAAG	AATAAGCTTAGTCCTCGGGGAATTCCT	This work
Check *PA2911*	AAGCGAGCGAAATCCTCTTC	GTGGTTCAGCAGAGACGATT	This work
*P. aeruginosa* RT-qPCR			
*PA14_73010*	CAGGAGCTGGTTTTCATCGG	GAATGCCCAGGTTCATCTCG	This work
*PA14_73000*	AGACGCTGGAGATGGAAGAG	CAAAAGAAGGCCTCGACCAG	This work
*PA14_39620*	ACCTGATCGACCTGTTCCTG	GGATTCTCGACCACCACCA	Michetti et al. (2025)
*dksA2*	GAAGCCCAGCAGGACTTCTTC	TGTCGAGCAGCTTCTTCTCCC	Mastropasqua et al. (2018)
*amiA*	GCTACAACGCCGACATGTTC	GGAGAGGGCATATACCGATG	(Michetti et al. (2025)
*rpmE2*	GCCGACGTGTACTTCCTGAT	GCGTCACGTAGGGATAGGTC	Mastropasqua et al. (2018)
*zrmA*	GACACCCGTATCGAGGACAT	GAAGCCACGGACGTTGTACT	Mastropasqua et al. (2018)
*rpsL*	GCTGCAAAACTGCCCGCAACG	ACCCGAGGTGTCCAGCGAACC	Michetti et al. (2025)

### RNA extraction, reverse transcription and real-time qPCR

Overnight cultures of *P. aeruginosa* PA14 were inoculated 1:1000 in VBMM, supplemented or not with ZnSO_4_, Co(NO_3_)_2_, CuSO_4_, NiSO_4_, or MnCl_2_ and grown overnight. Cultures were treated with RNAprotect (Qiagen) and total RNA was extracted. The RNA extraction was performed using the RNAeasy kit (Qiagen) according to the manufacturer’s protocol, with the addition of DNase (Qiagen) and lysozyme (Sigma-Aldrich). RNA concentration was determined with a NanoDrop™ Lite Spectrophotometer (Thermo Fisher Scientific). From each sample, 1 µg of RNA was reverse transcribed with the PrimeScript RT Reagent Kit and gDNA Eraser (Takara Bio Inc.). The primers used for RT-qPCR were designed using Primer3 (Table 2) (Koressaar and Remm 2007). RT-qPCR reactions were performed in triplicate in 10 µL reaction mixtures containing cDNA 50 ng, primers 0.3 µM, and 50% PowerUp SYBR Green Master Mix (Thermo Fisher Scientific). Amplifications were performed in a QuantStudio 3 real-time PCR system (Thermo Fisher Scientific) thermocycler with the following parameters: (i) initial denaturation at 95 °C for 4 min; (ii) 40 cycles of denaturation at 95 °C for 20 s, primer annealing at 60 °C for 30 s and extension at 72 °C for 30 s; (iii) melting curve, from 50 to 90 °C (rate: 0.58 °C every 5 s). The mRNA fold induction was calculated using the ΔΔCt method (Schmittgen and Livak 2008) and normalized to the *rpsL* housekeeping gene.

### Extracellular proteases assay

A 1:100 dilution of overnight-grown bacteria was inoculated into ASM and incubated overnight at 37 °C with shaking. To obtain culture supernatants, samples were centrifuged at 5000 × g for 15 min at 4 °C, and the supernatants were stored at − 20 °C until used. Before performing the protease assays, supernatants were treated with 50 µM TPEN, supplemented with 500 µM ZnSO_4_, Co(NO_3_)_2_ CuSO_4_, or MnCl_2_, or left untreated, and incubated overnight at 37 °C.

Elastolytic activity in culture supernatants was quantified using insoluble elastin Congo red, as described (Olson and Ohman 1992). An aliquot corresponding to 0.1 mL of each supernatant was added to 0.4 mL of 10 mM Tris–HCl, pH 8.0, containing 5 mg of substrate. The reaction mixture was incubated at 37 °C under shaking. After 24 h, undigested elastin was removed by centrifugation at 11,000 × g for 10 min, and the absorbance at 492 nm was measured using a microtiter plate reader (Sunrise Tecan).

The staphylolytic activity of supernatant samples of *P. aeruginosa* strains was determined by monitoring the decrease in absorbance at 595 nm of a heat-killed *Staphylococcus aureus* suspension (Caballero et al. 2001). *S. aureus* strain SH1000 (Table 1) was cultured overnight at 37 °C with shaking. Bacteria were pelleted by centrifugation, resuspended in 20 mM Tris–HCl, pH 8.8, to a final optical density at 595 nm of 1.0, and killed by heating at 100 °C for 30 min. Aliquots (0.1 mL) of supernatant were added to 0.9 mL of heat-killed bacterial suspension. Staphylolytic activity was determined by measuring the change in absorbance at 595 nm every 15 min for 3 h using a microtiter plate reader (Sunrise Tecan).

### Pyocyanin extraction and quantification

Overnight cultures of *P. aeruginosa* grown in E-VBMM with or without ZnSO_4_ or Co(NO_3_)_2_ (OD_600_ 2.0) were centrifuged at 5000 × g for 15 min, and the supernatants were filtered with a 0.22 µm filter and collected. Pyocyanin was extracted based on a previously established protocol, with minor modifications (Ingledew and Campbell 1969). Briefly, pyocyanin was extracted using chloroform, and the apolar fraction was recovered and treated with 10 mM HCl to transfer the pyocyanin into the aqueous phase. The acidified pink-red phase containing pyocyanin was recovered, and 0.1 N NaOH was added to restore the blue-pigmented form of pyocyanin. These steps were performed several times to enhance the purity of the pyocyanin. The acidified pink-red phase was collected, and its absorbance was analyzed within the 400–600 nm range. The concentration of pyocyanin was calculated using the molar extinction coefficient Ɛ (HCl [0.2 N]) = 2.46 × 10^3^ M⁻^1^ cm⁻^1^, by the formula c = A_520_/Ɛ, where c is the molar concentration, and A_520_ is the absorbance peak of pyocyanin. Each experiment was performed on three independent colonies and was repeated three times.

### *fliE* promoter activity

The reporter plasmid p*fliE*pTZ110 (Table 1), carrying the promoter region of *fliE* upstream of the lacZ gene, was mobilized from *E. coli* DH5α into PA14 wild-type and *znuAzrmB* mutant strains by triparental mating, using *E. coli* HB101 pRK2013 as the helper strain (Table 1). The exconjugants were pre-inoculated overnight in LB medium, diluted 1:1000 in E-VBMM complemented or not with metals, and incubated at 37 °C for 18 h. Three independent colonies were tested for beta-galactosidase activity for each experimental condition, as previously described (Ammendola et al. 2022). Absorbance was measured by a microplate reader (Sunrise, Tecan).

### Analyses of swarming motility

*P. aeruginosa* swarming motility was assayed following a protocol described elsewhere with minor modifications (Coleman et al. 2020). Swarming plates were prepared with 0.4% agar BM2 glucose minimal medium, without (NH_4_)_2_SO_4_, and with 10 µM EDTA. Briefly, overnight LB inocula were diluted 1:50 in fresh LB medium and grown to exponential phase (approximately OD_600_ = 0.3). Aliquots corresponding to 5 µL were spotted in the center of swarming plates supplemented or not with 10 µM ZnSO_4_, Co(NO_3_)_2_, CuSO_4_, or MnCl_2 2_ and incubated upside up at 37 °C. The plates were imaged after 18 h on a ChemiDoc imaging system (Bio-Rad).

### Hydrogen peroxide tolerance assay

Overnight grown bacteria were inoculated 1:1000 in E-VBMM supplemented or not with 5 µM ZnSO_4_ or Co(NO_3_)_2_ and incubated at 37 °C overnight with shaking. Bacteria were then diluted to 10^6^ CFU mL^−1^ in sterile PBS, and each sample was divided into aliquots, some of which were exposed to 0.5 or 0.75 mmol L^−1^ H_2_O_2_ (Merck) and others were untreated as a control. The samples were incubated at 37 °C. After 1 h, 1000 units of catalase (Sigma Aldrich) were added to inactivate the hydrogen peroxide, and serial dilutions of bacteria were plated on LB-agar for Colony Forming Unit (CFU) counting. The number of colonies in each spot was quantified using an automatic colony counter (Scan500 ®, Interscience). The survival percentage was calculated by the formula: (CFU of exposed bacteria / CFU of unexposed bacteria) × 100.

### *P. aeruginosa PA2911* mutant construction

The *PA2911* deletion mutant was obtained using the gene replacement method (Hoang et al. 1998) with minor modifications (D’Orazio et al. 2015). The gentamicin resistance cassette was obtained by BamHI (New England Biolabs) digestion of plasmid pSP856 (Table 1). The 5’ and the 3’ terminal fragments of *PA2911* were amplified using PA14 DNA as a template and with the primers listed in Table 2. The EcoRI/BamHI and BamHI/HindIII (New England Biolabs) digested 5' and 3' fragments and the gentamicin resistance cassette were cloned in plasmid pEX18Tc, and the resulting plasmid was mobilized to PA14 wild-type by tri-parental mating as already described (D’Orazio et al. 2015). The *PA2911* deletion in PA14 was confirmed by PCR using the primers in Table 2.

### *P. aeruginosa *competition assays in *G. mellonella*

Fourth-instar stage *G. mellonella* larvae (approximately 400 mg each) were purchased from a local vendor and inoculated on the same day. PA14 wild-type and mutant strains were grown in LB to an OD_600_ of 0.6–0.8, diluted in PBS (pH 6.4) to ~ 2500 CFU/mL, mixed in a 1:1 ratio, and injected into the larvae (10 µL per mixture) using a Hamilton syringe, as previously described (Michetti et al. 2025). Input ratios were confirmed by plating on PIA and replica plating on gentamicin-supplemented PIA. When the larvae began to show signs of illness (18–24 h post-inoculation), 10 μL of hemolymph containing the bacterial output was collected by pricking the abdomen with a sterile needle. Serial dilutions of the collected hemolymph were plated on PIA and incubated overnight at 37 °C. The following day, at least 100 colonies from each larva were replica plated on gentamicin-supplemented PIA plates to assess the strain A/strain B ratio in the outputs. Each competitive index (CI) was calculated using the formula *CI* = *output (strain A/strain B)/input (strain A/strain B)*.

### Statistical analyses

Statistical analyses were performed using the GraphPad software (v. 10.1.1). Depending on the experimental design, the following tests were applied as specified in the figure captions: two-way ANOVA followed by Tukey’s, Sidak’s, or uncorrected Fisher’s LSD multiple comparisons test; one-way ANOVA followed by Sidak’s or Bonferroni’s multiple comparisons test; and unpaired Student’s t-test.

## Results

### Cobalt accumulation in *P. aeruginosa* is dependent on Zinc availability

Previous results have shown that, in Zn-restricted environments, *P. aeruginosa* strains impaired in Zn uptake increase their intracellular Co content and that Co supplementation enhances their growth (Mastropasqua et al. 2017; Secli et al. 2024; D’Orazio et al. 2015; Pederick et al. 2015). To further investigate the role of Co in the response of *P. aeruginosa* to Zn deficiency, we compared the intracellular Zn and Co content in PA14 wild-type and a *znuA* mutant strain following their growth under varying Zn supplementation conditions.

**Fig. 1:**
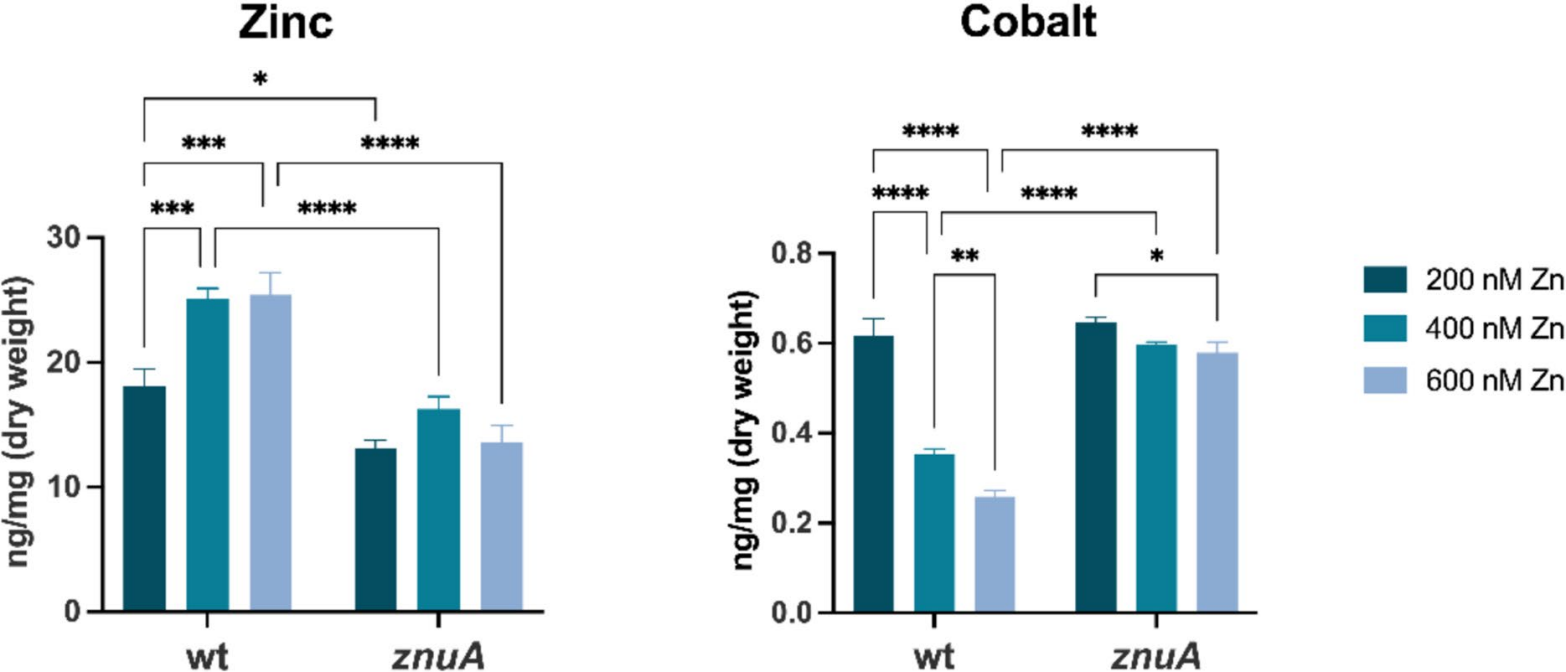
Intracellular Zn and Co content in *P. aeruginosa*. ICP-MS analyses of bacteria grown in E-VBMM supplemented with trace metals (0.1 μM FeSO_4_, NiSO_4_, Co(NO_3_)_2_, CuSO_4,_ and MnCl_2_) and increasing ZnSO_4_ concentrations, as indicated in the legend. Bars are the mean values of three biological replicates ± SD. Statistical analyses were performed using two-way ANOVA and Tukey’s multiple comparisons test. Asterisks indicate statistically significant differences between wild-type and *znuA* strains (*p < 0.05; **p < 0.01; ***p < 0.001; ****p < 0.0001)

The strains were grown in E-VBMM supplemented with trace amounts of transition metals, as previously described (Mastropasqua et al. 2017), with variable concentrations of ZnSO_4_ (200, 400, or 600 nM). As shown in Fig. 1, intracellular Zn levels in the PA14 wild-type strain increase with Zn availability in the growth medium. Noticeably, intracellular Co diminishes in a dose-dependent manner as Zn availability increases. In the *znuA* mutant strain grown with 200 nM Zn, intracellular Zn levels are lower than in PA14 wild-type and do not significantly change with increasing Zn availability, confirming the primary role of the ZnuABC system in Zn uptake. Conversely, the intracellular Co levels in the *znuA* mutant, comparable to those in PA14 wild-type in 200 nM Zn conditions, remain high even with Zn supplementation, showing only a slight decrease at 600 nM Zn. The differences in intracellular Co levels between the *znuA* mutant and the PA14 wild-type in the presence of 400 or 600 nM Zn support the hypothesis that *P. aeruginosa* imports Co when intracellular Zn levels are low. It is worth noting that ICP-MS analysis revealed that other metals also show differences in their intracellular concentrations, depending on both the genotype (wild type or *znuA*) and the concentration of Zn in the growth medium (Supplementary Fig. 1). However, none of these metals exhibit a trend comparable to that observed for Co.

### Cobalt represses the Zur-regulon by interacting with Zur

**Fig. 2:**
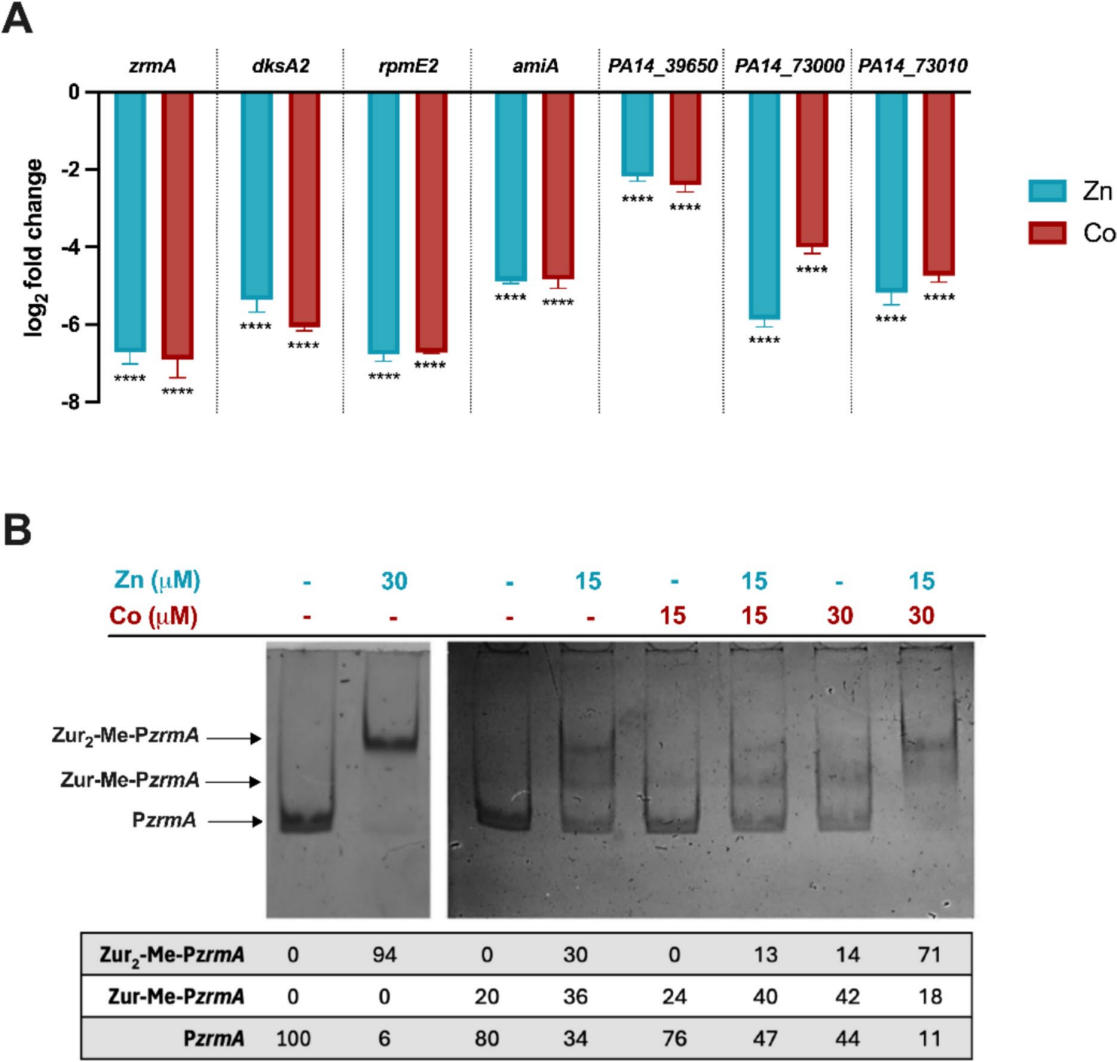
**A** mRNA expression levels of Zur-regulated genes. qRT-PCR on Zur-regulated genes from *P. aeruginosa* PA14 wild-type grown in VBMM supplemented or not with 2 µM ZnSO_4_ or 2 µM Co(NO_3_)_2_. Data are mean values ± S.D. of triplicates and expressed as Relative fold expression (log_2_ΔΔCt) compared to the gene expression in VBMM without metals (control). Statistical analyses were performed using one-way ANOVA and Sidak's multiple comparison test. Asterisks indicate statistically significant differences between each sample and the control (****p < 0.0001). **B** EMSA of *zrmA* promoter and Zur*.* DNA (30 ng) was mixed with Zur 1 µM and TPEN 30 µM. Each mixture was incubated with different concentrations of ZnSO_4_ and CoCl_2_ or left untreated, as indicated. Reactions were loaded on non-denaturing 7.5% polyacrylamide gels, stained with ethidium bromide, and viewed under UV light. The figure included in Panel B shows a representative result from experiments performed several times with consistent outcomes

Gene expression analyses have already indicated that the transcription of *znuA* and *zrmA* is downregulated by Co under Zn deficiency conditions, suggesting a role for Co in modulating regulatory pathways associated with Zn uptake (Mastropasqua et al. 2017). Since the Zur-regulon includes genes beyond those directly involved in Zn uptake, we expanded the analyses by examining the transcriptional activity of other Zur-dependent genes. These include *dksA2,* which encodes a Zn-independent structural paralog of the transcription factor DksA (Furman et al. 2013), *rpmE2*, which encodes the ribosomal Zn-independent protein L31p that substitutes the Zn-containing paralog under Zn starvation (Pederick et al. 2015), *amiA*, which encodes a Zn-dependent N-acetylmuramoyl-L-alanine amidase (Michetti et al. 2025), *PA14_39620* (*PA1925* in *P. aeruginosa* PAO1), encoding for an uncharacterized protein, and *PA14_73000* and *PA14_73010* (*PA5534* and *PA5535* in *P. aeruginosa* PAO1, respectively), encoding for two annotated metallochaperones with unknown functions (Pederick et al. 2015). Figure 2A shows that Co represses all the selected genes, suggesting that the transcription of the whole Zur-regulon is downregulated in Zn-starved bacteria accumulating Co ions. Other metals, such as Cu, Ni, or Mn, are unable to repress the expression of Zur-regulated genes (Supplementary Figs. 2 and 3). To test whether Co can substitute for Zn by directly interacting with the Zur transcription factor, we purified the *P. aeruginosa* Zur protein and performed EMSA of the *zrmA* promoter. The assays included the Zn-chelating agent TPEN to ensure a proper demetallation of purified Zur. Control experiments involving the use of holo-Zur, of a non-specific promoter or of denatured Zur in presence or absence of metals are included in Supplementary Fig. 4A–C. Figure 2B demonstrates that the incubation of apo-Zur with 30 µM Zn triggers a complete band shift, meaning that all the DNA fragments are bound to the protein. In the presence of 15 µM Zn, multiple bands appear, likely corresponding to the unbound promoter (P*zrmA*) and the DNA interacting with the Zur monomers or dimers (indicated as Zur-Me-P*zrmA* and Zur_2_-Me-P*zrmA*, respectively). In contrast, 15 µM Co alone or with an equimolar concentration of Zn did not cause a DNA shift. However, increasing Co concentration to 30 µM Co triggers a shift comparable to that of the 15 µM Zn-treated sample, and a complete DNA-Zur interaction was observed when a 2(Co):1(Zn) ratio was employed (i.e., 15 µM Zn and 30 µM Co). Similar results could not be replicated using Cu in place of Co (Supplementary Fig. 4D). These findings suggest that Co can partially substitute Zn in *P. aeruginosa* Zur, promoting the interaction with the target promoter.

### Cobalt can bind the *P. aeruginosa* extracellular Zn-dependent proteases

**Fig. 3:**
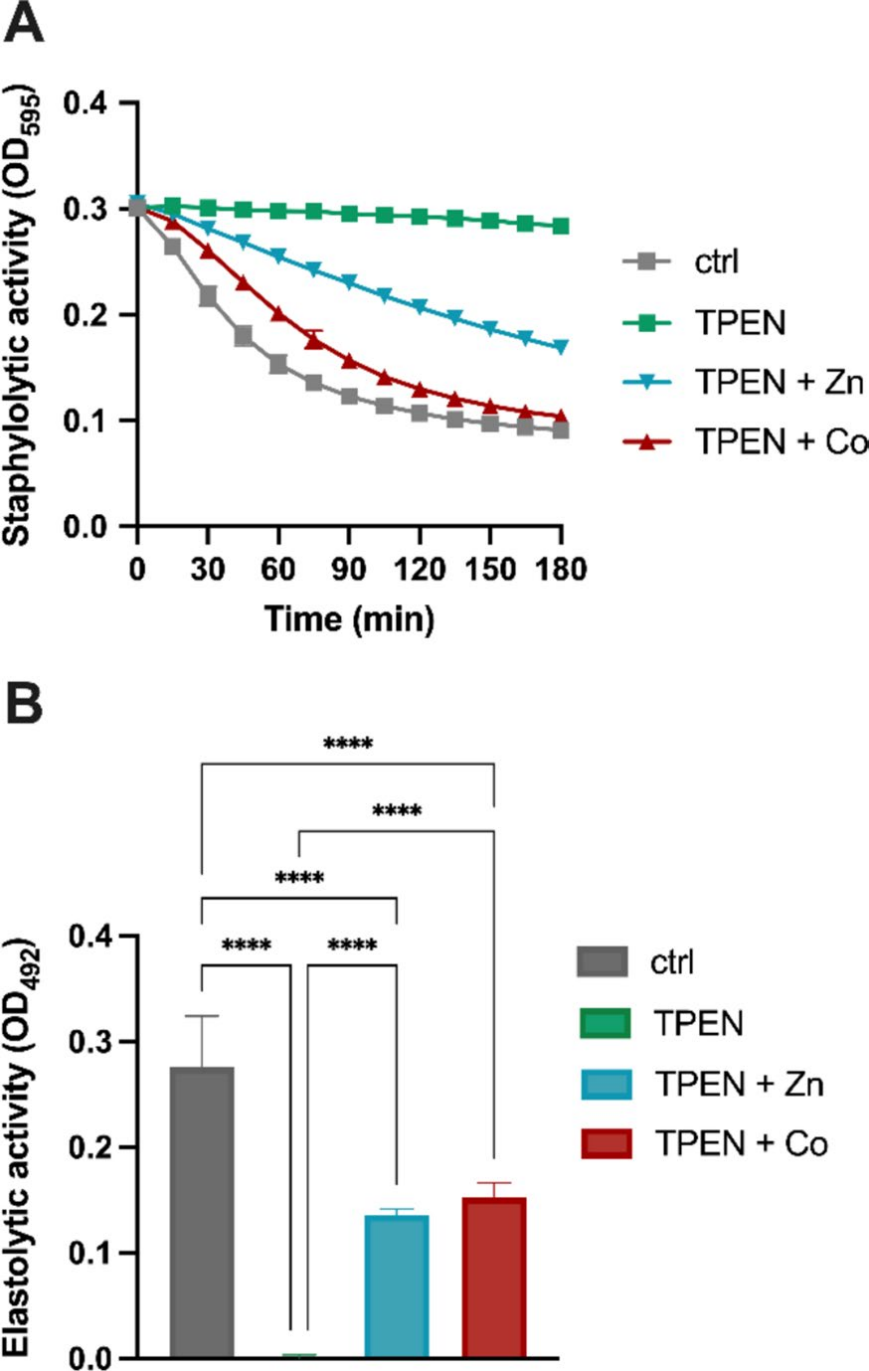
Extracellular protease activity of *P. aeruginosa*. Staphylolytic (**A**) and elastolytic (**B**) activities were assessed in the supernatant collected from PA14 wild-type grown overnight in ASM. The supernatants were treated with 50 µM TPEN, with or without the addition of 500 µM ZnSO_4_ or Co(NO_3_)_2_, or left untreated (ctrl). Data represent mean values from three independent colonies ± S.D. Statistical analysis was conducted using one-way ANOVA and Bonferroni's multiple comparisons test. Asterisks denote statistically significant differences (****p < 0.0001)

To explore whether *P. aeruginosa* can use Co to replace Zn in virulence-related functions, we examined the potential of this metal to restore the staphylolytic and elastolytic activity of the extracellular proteases LasA and LasB. Several studies have highlighted that these Zn-dependent proteases exhibit poor activity when *P. aeruginosa* is grown in Zn-limited conditions (Olson and Ohman 1992; Matsumoto 2004; D’Orazio et al. 2015; Mastropasqua et al. 2017; Vermilyea et al. 2021). To this aim, we collected the supernatant of *P. aeruginosa* PA14 wild-type grown in ASM, treated it with the metal chelator TPEN, and assayed for extracellular protease activity after supplementing the samples with Zn, Co, Cu or Mn. Figure 3 shows that in this medium, *P. aeruginosa* produced highly active staphylolytic and elastolytic proteases. However, the treatment of the supernatant with TPEN strongly reduced the activity of both proteases, likely due to the removal of Zn from the active site of the enzymes. As shown in the results, both Co and Zn partially restored the activity of the TPEN-treated enzymes. Notably, Co enhanced LasA activity beyond the levels observed with Zn supplementation (Fig. 3A), suggesting differences in catalytic efficiency or metal-binding affinities. This finding indicates that *P. aeruginosa* may exploit Co to maintain the activity of Zn-dependent virulence factors under Zn-limited conditions. In contrast, Mn failed to restore the activity of LasA and LasB, whereas Cu unexpectedly restored full catalytic activity to LasA, but not to LasB (Supplementary Fig. 5A). While most Zn metalloproteinases lose activity upon metal substitution, exceptions exist, such as dipeptidyl peptidase III (DPP III), which retains full activity with Cu (Fukasawa et al. 2011).

### Cobalt restores the impaired motility of Zn-starved *P. aeruginosa*

**Fig. 4:**
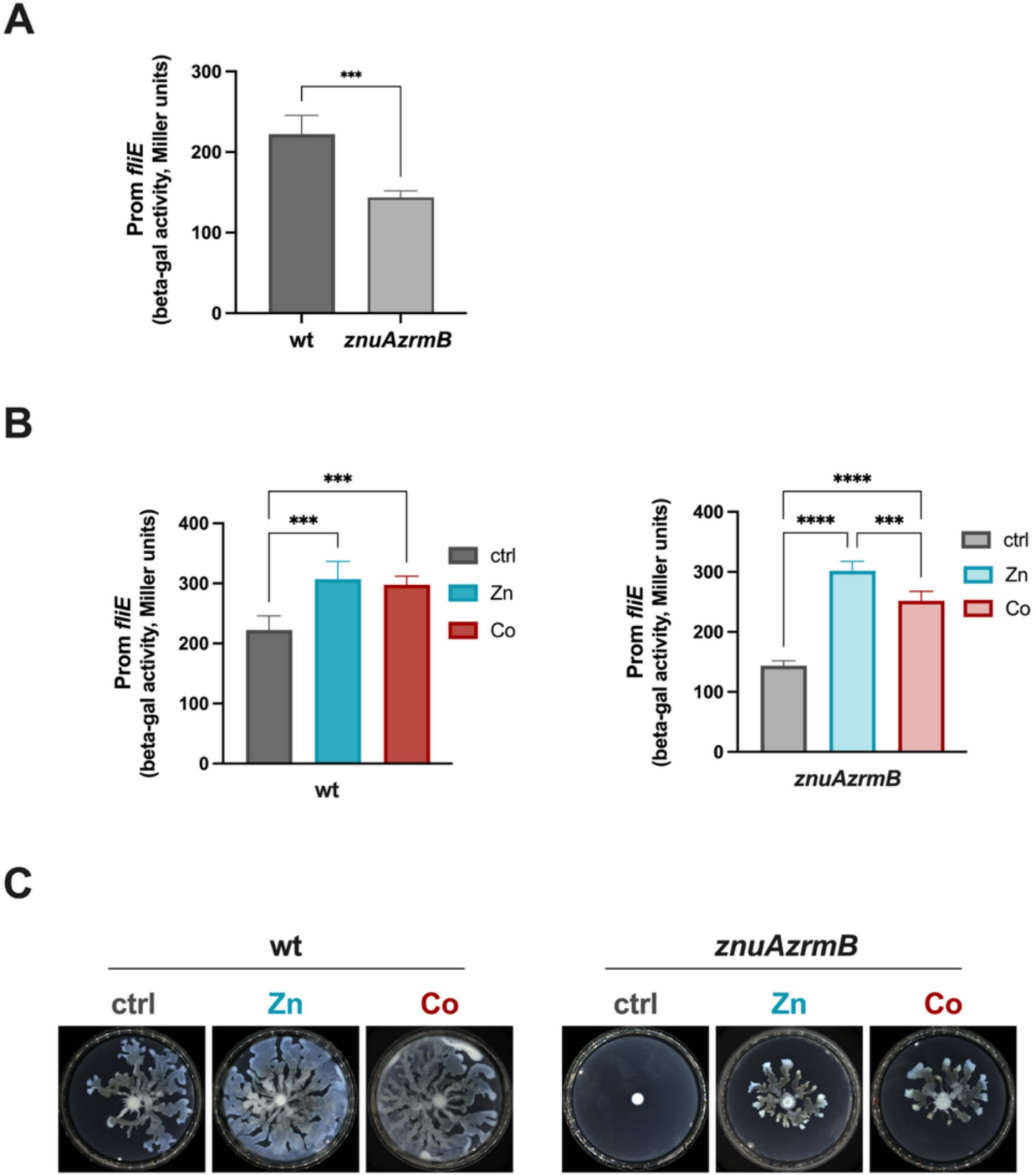
Analyses of P*. aeruginosa* motility. **A** Comparison of the *fliE* promoter activity between the PA14 wild-type and *znuAzrmB* mutant strain, grown in E-VBMM. **B** The same values were compared to the activity of the *fliE* promoter in PA14 wild-type and *znuAzrmB* mutant strains grown in E-VBMM supplemented with 7 µM ZnSO_4_ or Co(NO_3_)_2_, as indicated in the legends. Data are the mean value of a triplicate ± SD. Statistical analyses were performed by the unpaired Student t-test (panel A, ***p < 0.0005) or one-way ANOVA and Tukey's multiple comparison tests (panel B, ***p < 0.0005; ****p < 0.0001). (C) Images of swarming plates of PA14 wild-type and *znuAzrmB* mutant strain, containing 10 µM EDTA (ctrl) supplemented with 10 µM ZnSO_4_ or 10 µM Co(NO_3_)_2_. The experiment was repeated three times, with similar results

It has been previously shown that Zn availability strongly affects bacterial motility in different bacterial species, including *P. aeruginosa* (Ammendola et al. 2016; Mastropasqua et al. 2018). In particular, Zn starvation reduces motility in *P. aeruginosa*, as observed in the *znuAzrmA* mutant strain grown in Zn-restricted conditions. This motility defect can be rescued by adding Zn, but not by Fe. To assess whether Co could also restore the motility defect caused by Zn paucity, we analyzed the activity of the *fliE* promoter and the swarming motility of the PA14 wild-type and *znuAzrmB* mutant strains. Figure 4A shows that the *fliE* promoter activity is downregulated in the *znuAzrmB* mutant strain compared to the PA14 wild-type, but is significantly increased upon supplementation with either Zn or Co in both strains (Fig. 4B). Accordingly, the swarming defect observed in the *znuAzrmB* mutant strain on EDTA-supplemented plates is rescued by adding either Zn or Co (Fig. 4C). Mn and Cu were unable to rescue the swarming defect (Supplementary Fig. 5B).

### Cobalt affects the production of pyocyanin

**Fig. 5:**
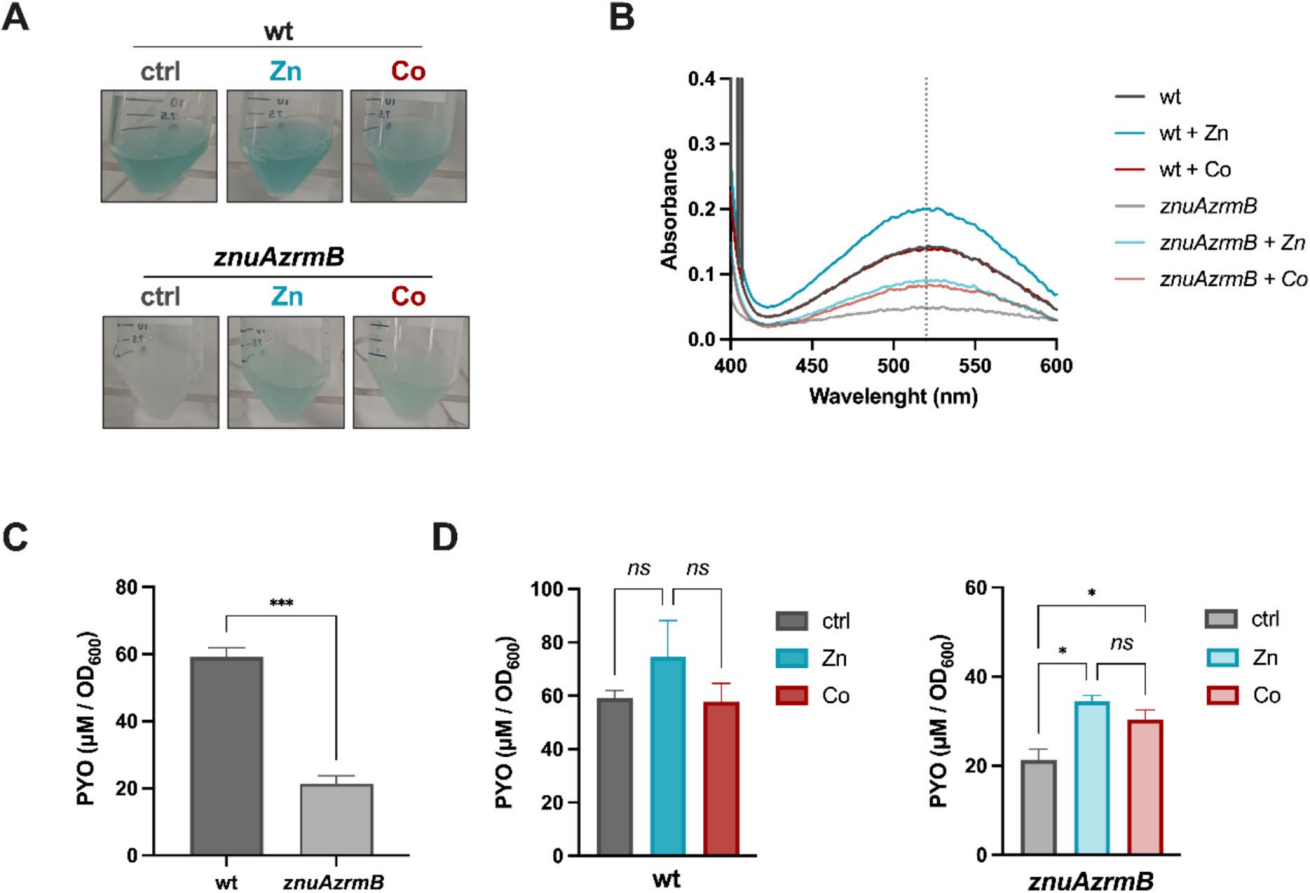
Pyocyanin production in *P. aeruginosa* wild-type and *znuAzrmB* mutant. **A** Images of PA14 wild-type and *znuAzrmB* cultures grown overnight in E-VBMM supplemented or not with 5 µM ZnSO_4_ or Co(NO_3)2_. **B** Absorbance spectra (400–600 nm) of pyocyanin extracted from the culture supernatants. A dotted vertical line indicates the peak of pyocyanin at λ = 520 nm. **C** Comparison of pyocyanin concentration, normalized based on the optical density of the cultures (OD_600_), between the PA14 wild-type and *znuAzrmB* mutant strains. **D** The same values were compared to those from Zn and Co-supplemented growth of PA14 wild-type and *znuAzrmB* mutant strains. Statistical significances were calculated using the unpaired Student's t-test (panel C, ***p < 0.005) and the One-Way Ordinary ANOVA test (panel D, *p < 0.05; *ns*, not significant)

Pyocyanin is a blue-green, redox-active small molecule secreted by *P. aeruginosa*, which contributes to tissue injuries and epithelial cell killing in the CF lung (Dietrich et al. 2006). Differences in the pigmentation of Zn-starved liquid cultures of the *P. aeruginosa* PA14 wild-type and strains lacking the main Zn-importers have been previously reported and related to differences in the secreted pigments profile (Mastropasqua et al. 2017; Secli et al. 2024). Purifying pyocyanin from culture supernatants revealed that the *zrmAzrmB* mutant strain produces a lower amount of this pigment than the PA14 wild-type strain when grown in a Zn-restricted medium. Notably, Co supplementation restores pyocyanin production in the *znuAzrmB* strain to levels comparable to those achieved with Zn (Fig. 5). Interestingly, while adding Zn to the growth medium significantly enhanced pyocyanin production in both strains, Co only increased pyocyanin production in the Zn-starved mutant, with no effect observed in the wild-type strain.

### Cobalt protects Zn-starved *P. aeruginosa* from oxidative stress

**Fig. 6:**
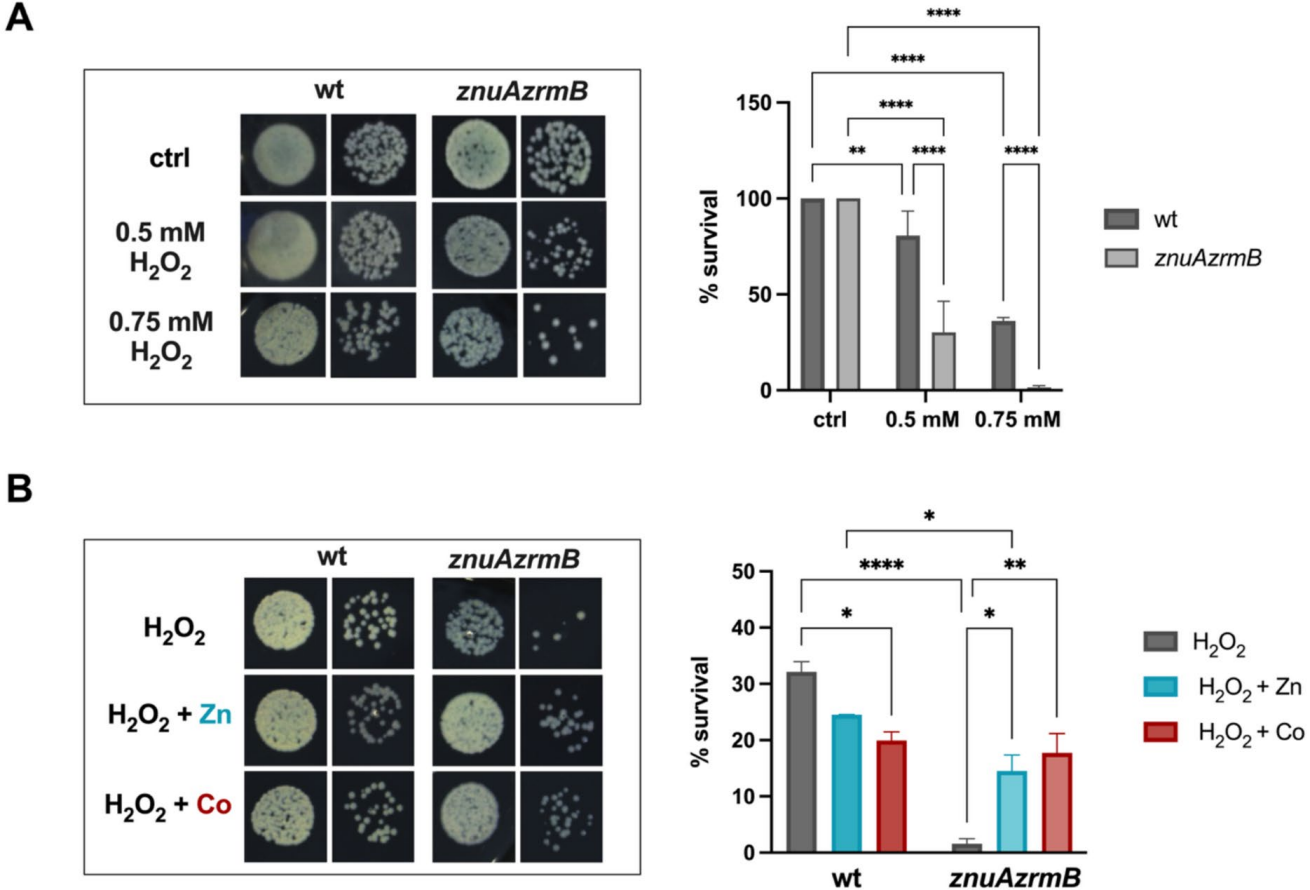
Sensitivity of *P. aeruginosa* to H_2_O_2_. **A** Images of serial dilutions of the PA14 wild-type and *znuAzrmB* strains spotted on LB plates after exposure to varying amounts of H_2_O_2_, as detailed in Materials and Methods (left panel). The survival percentage was calculated by considering the number of CFUs in untreated samples as 100% (right panel). Values represent the mean of three biological replicates ± SD. Statistical analysis was conducted using Two-way ANOVA and Uncorrected Fisher's LSD test (**p < 0.005; ****p < 0.0001). **B** The PA14 wild-type and *znuAzrmB* strains were cultured in E-VBMM, with or without 5 µM ZnSO_4_ or Co(NO_3_)_2_, and exposed to 0.75 mM H_2_O_2_. The left panel displays a representative image of the serial dilutions of the treated samples spotted on LB agar plates. The right panel illustrates the survival rates, calculated as described above. Statistical analyses were performed using Two-way ANOVA and Sidak’s multiple comparisons test (*p < 0.05; **p < 0.005; ****p < 0.0001)

Numerous studies have highlighted the importance of Zn uptake in bacterial resistance to oxidative stress and that inactivation of Zn transporters increases bacterial susceptibility to H₂O₂-induced damage (Gaballa and Helmann 2002; Cerasi et al. 2014). To explore the role of Zn in *P. aeruginosa*, we exposed PA14 wild-type and *znuAzrmB* strains to hydrogen peroxide for one hour and compared their survival rates to unexposed controls. Figure 6A shows the effect of two concentrations of H_2_O_2_ on PA14 wild-type and *znuAzrmB* strains grown in a Zn-restricted medium. Even at the lowest dose (i.e., 0.5 mM H_2_O_2_), the *znuAzrmB* mutant exhibited a higher sensitivity than the PA14 wild-type, which was only slightly affected. Exposure to 0.75 mM H₂O₂ had a pronounced effect on both strains, almost entirely hampering the survival of the *znuAzrmB* mutant. These results confirm that Zn starvation increases susceptibility to oxidative stress in *P. aeruginosa*. To determine whether Co, like Zn, provides a protective effect, we exposed the strains grown in E-VBMM supplemented with metals to H_2_O_2_. As shown in Fig. 6B, Zn-supplementation did not significantly affect the wild-type tolerance to H₂O₂, whereas the presence of Co in the growth medium slightly increased its sensitivity. Conversely, the hypersensitivity of the *znuAzrmB* mutant to H₂O₂ was significantly rescued by supplementation with either Zn or Co to levels close to those of the metal-supplemented PA14 wild-type.

### PCH-mediated uptake of cobalt favors *P. aeruginosa* colonization of *G. mellonella*

We recently demonstrated that PCH mediates Co trafficking during Zn starvation, through transporters encoded by the Zur-regulated operons *PA2911-2914* and *PA4043-4066* (Secli et al. 2024). Using the *G. mellonella* infection model, which we proved to be a reliable platform for studying *P. aeruginosa* responses to Zn starvation in the context of host nutritional immunity (Michetti et al. 2025), we investigated whether impairments in the PCH-Co transport systems might affect the ability of *P. aeruginosa* to colonize *G. mellonella*.

**Fig. 7:**
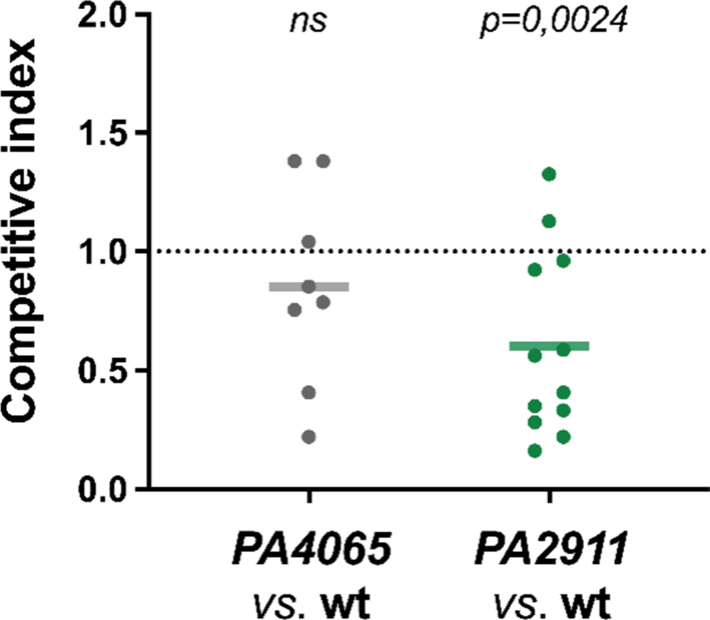
Competition assays in *G. mellonella*. Mixed inocula in a 1:1 ratio of PA14 wild-type and *PA4065* deleted strains (gray dots) or PA14 wild-type and *PA2911* deleted strains (green dots) were inoculated into *G. mellonella* larvae. Each dot represents the competitive index in one larva, calculated using the formula reported in the Materials and Methods, assuming the mutant strain as strain A and the wild-type strain as strain B. Median values of the competitive index are shown by a horizontal line, and statistical differences between inputs and outputs are assessed using the Student’s t-test

Competition assays between the *PA2911* mutant and the PA14 wild-type strains revealed that inactivating the PCH-Co entry route significantly affected the fitness of *P. aeruginosa* facing Zn restriction in the colonized host. In contrast, no significant differences in colonization ability were observed between the *PA4065* mutant and the PA14 wild-type strain (Fig. 7). This result suggests a role for Co uptake through PCH in alleviating Zn starvation caused by the host nutritional immunity response.

## Discussion

*P. aeruginosa* can withstand host-imposed Zn starvation using multiple highly efficient pathways to capture and utilize Zn from the infected tissues. These mechanisms, transcriptionally controlled by Zur, include (i) the activity of the ZnuABC high-affinity Zn importer, widely conserved among Gram-negative bacteria, (ii) the synthesis, secretion, and recapturing of the metallophore pseudopaline, a strategy limited to a few bacteria and unique to *P. aeruginosa* among *Pseudomonas* species, and (iii) the intracellular redistribution of available Zn through the use of Zn-independent protein variants to sustain other strictly Zn-dependent metabolic functions (Blaby-Haas et al. 2011; Pederick et al. 2015). The high expression of Zur-regulated genes supports the importance of proper Zn acquisition in the host environment during *P. aeruginosa* infections in humans (Cornforth et al. 2018) and by the evidence that a failure in Zn uptake causes the impairment of several virulence-related traits and a significant loss of the ability to persist in different animal models (D’Orazio et al. 2015; Mastropasqua et al. 2018; Gonzalez et al. 2019; Secli et al. 2023; Michetti et al. 2025). Our previous research found that a *P. aeruginosa* strain impaired in Zn import, i.e., a *znuA* deletion mutant, accumulates Co intracellularly, that Co may promote growth of a *znuAzrmB* mutant, and that two previously uncharacterized Zur-regulated operons are involved in PCH-mediated Co trafficking during Zn starvation (D’Orazio et al. 2015; Mastropasqua et al. 2017; Secli et al. 2024). These observations suggested that Co may serve as a compensatory strategy, allowing *P. aeruginosa* to mitigate severe Zn starvation by substituting the metal in some Zn-dependent proteins.

The substitution of Zn with the spectroscopically active Co is often exploited in techniques for studying catalytic and structural properties of enzymes where Zn is the native metal cofactor. Co and Zn share a similar atomic radius and coordination chemistry, and most Co-substituted enzymes retain their activity (Maret and Vallee 1993). It is broadly assumed that the use of Co in vivo is limited, mainly as a cofactor in cobalamin and a few other examples of non-corrin enzymes. For this reason, import systems dedicated to Co were considered limited to microorganisms that synthesize cobalamin de novo. At the same time, export systems must ensure that Co concentration inside the cell does not exceed the physiological requirement. Bacterial tolerance to Co can vary greatly among microorganisms, and its toxicity is mainly a consequence of enzyme mismetallation, Fe-S cluster inactivation, and possibly the generation of ROS through the Fenton reaction (Osman et al. 2021). *P. aeruginosa* has a relatively low tolerance toward Co compared to other human pathogens (Fantino et al. 2010; Ammendola et al. 2020; Schürmann et al. 2024). A Co concentration above 5 µM causes a marked growth delay when this microorganism is grown in a chemically defined medium. Still, interestingly, the same Co supplementation restores the growth defect caused by Zn starvation (Mastropasqua et al. 2017; Secli et al. 2024).

The results reported in this study confirm a connection between the homeostasis of the two metals, as revealed by an opposite accumulation trend in media with variable Zn content (Fig. 1). Interestingly, this pattern is deregulated in the *znuA* mutant, which is less capable of importing Zn and maintains intracellular Co levels comparable to those of the PA14 wild type grown with the lowest Zn concentration. As a consequence of Co uptake, the expression of a subset of Zur-regulated genes was markedly downregulated in *P. aeruginosa* wild-type (Fig. 2A). This observation raised the hypothesis that Co may directly modulate gene expression by interacting with Zur. The Zn-uptake regulator Zur is a dimeric protein, with each subunit containing both a structural binding site coordinated by four cysteine residues and a regulatory site, where the metal is bound with weaker affinity (Outten et al. 2001). While it has been reported that in *Salmonella enterica*, Zur can bind up to four Co ions and interact with the *znuA* promoter with an affinity comparable to the Zn-containing Zur (Osman et al. 2017), a recent study has shown that Co can bind the regulatory site of the *Acinetobacter baumannii* Zur protein, but not the structural site (Kim et al. 2024). Here, we have shown that purified *P. aeruginosa* Zur binds the *zrmA* promoter when loaded with Zn and that a similar effect can be triggered by Co only if Zn is also present in the reaction mixture, in a Co:Zn ratio of 2:1 (Fig. 2B). On the one hand, this result indicates that Co may modulate gene expression directly via interaction with Zur. On the other hand, it highlights that Zn and Co are not entirely interchangeable. In analogy to what has been suggested for *A. baumannii*, we can hypothesize that incorporating Zn in the Zur structural site is necessary for DNA binding and cannot be substituted by Co (Kim et al. 2024).

As observed with Zur, other Zn-cofactored proteins may incorporate Co, thus favoring bacterial adaptation to Zn-limiting conditions. Notably, several Zur-regulated genes are annotated as being involved in Co homeostasis. Examples include the *PA14_39640* (*cobN*) gene, which is part of the Zur-regulated *PA14_39620*- *PA14_39650* operon (*PA1922-PA1925* in *P. aeruginosa* PAO1), and it is annotated as the cobaltochelatase CobN subunit (Winsor et al. 2016). Additionally, PA14_73000 and PA14_73010 encode two G3E GTPase COG0523-like metallochaperones, showing similarities with proteins associated with cobalamin biosynthesis. Several studies have demonstrated that this family of proteins, conserved across prokaryotes and eukaryotes, plays a role in Zn allocation to specific metalloproteins (Sydor et al. 2013; Weiss et al. 2022). Among their targets, Weiss et al. identified the methionine aminopeptidase, a protein known to be able to incorporate Co (Kobayashi and Shimizu 1999). This observation leads to the intriguing speculation that these proteins may also facilitate the redistribution and allocation of Zn and Co to various metalloproteins, enabling an adaptive response to reduced intracellular Zn availability.

The staphylolytic LasA protease and the elastolytic LasB protease are key virulence factors contributing to host tissue degradation, thus promoting *P. aeruginosa* colonization and persistence in CF airways (Cowell et al. 2003; Kessler et al. 2004; Everett and Davies 2021). Both enzymes need Zn for their catalytic function, and Zn-starvation and the cultivation of bacteria in the presence of calprotectin significantly reduce their activity (D’Orazio et al. 2015; Vermilyea et al. 2021). Consistently, our results show that the treatment with the Zn chelator TPEN strongly reduced the activity of these extracellular proteases. Interestingly, the increased activity observed in the presence of Zn or Co suggests that these proteases possess a degree of metal-binding site promiscuity in vitro, allowing functional substitution of Zn with Co. We also investigated the effect of Co on pyocyanin production, a virulence-associated phenazine metabolite produced by *P. aeruginosa* during infection. Pyocyanin participates in host tissue damage by promoting the generation of reactive oxygen species that contribute to epithelial cell injury in CF lungs (Dietrich et al. 2006). The production of phenazine metabolites, including pyocyanin, is generally reduced under metal-starvation conditions, such as the presence of calprotectin (Zygiel et al. 2019). Consistently, our results demonstrate a pronounced reduction in pyocyanin levels in the *znuAzrmB* mutant compared to the PA14 wild-type strain. Interestingly, while Zn supplementation enhanced pyocyanin production in both strains, Co only restored pyocyanin synthesis in the *znuAzrmB* mutant, with no observable effect in the wild-type. This supports the idea that Co plays a compensatory role specifically under conditions of Zn deficiency, with its uptake and efflux being tightly regulated (Secli et al. 2024). Alternatively, Co may influence pyocyanin production in a concentration-dependent manner, exhibiting a threshold beyond which it either promotes or inhibits its production. A recent study supports a dose-dependent metal effect on pyocyanin production, showing that Zn oxide nanoparticles stimulate its synthesis at low concentrations and inhibit it when present in excess (Honselmann Genannt Humme et al. 2024). Consistently, high levels of Zn have been shown to repress pyocyanin production via the CzcR regulator (Dieppois et al. 2012; Liu et al. 2022).

Another key aspect of *P. aeruginosa* pathophysiology is its ability to withstand host-generated reactive oxygen species (ROS), increasing antioxidant responses often dependent on essential metals availability, including Zn (Gaballa and Helmann 2002; Cerasi et al. 2014). Consistently, our results show that the *znuAzrmB* mutant exhibited a marked hypersensitivity to H₂O_2_. Supplementation with either Zn or Co significantly alleviated this sensitivity, suggesting that Co can functionally compensate for Zn in supporting the antioxidant response. A protective role for Co has also been reported in *S. enterica*, where Co supplementation in Zn-deficient growth media restored the resistance of *znuABC* mutants to H₂O₂ and the activity of the Zn-cofactored superoxide dismutase SodCII (Ammendola et al. 2020). Since *P. aeruginosa* lacks Zn-dependent antioxidant enzymes, the protective role of Co supplementation may involve the formation of coordination complexes with thiol groups in cysteine, leading to the stabilization and protection of proteins from oxidative damage. However, the reduced survival of the wild-type strain exposed to H₂O₂ in the presence of Co suggests that, if Zn is present, Co exerts cytotoxic effects, highlighting, once again, that these two metals are not entirely interchangeable.

Some critical differences in the properties of Co and Zn contribute to the preference for Zn as a protein cofactor. Zn is redox-inactive, stably maintaining its divalent state, whereas Co can switch between the + 2 and + 3 oxidation states, potentially forming reactive chemical species that increase oxidative damage (Angelé-Martínez et al. 2023). The redox properties of this metal, together with its low environmental abundance, provide a likely evolutionary rationale for the broader use of Zn over Co, and help explain why organisms cannot sustain high intracellular levels of Co and must maintain a tightly regulated balance between its uptake and export. The low abundance of Co compared to Zn in living systems raises the question of whether the availability of Co in human tissues is sufficient to enable *P. aeruginosa* to compensate for Zn starvation. In living systems, most metals are tightly bound to proteins, and the actual availability of these metals for microorganisms remains largely unclear. For instance, while the intracellular concentration of Zn in most cell types is around 0.2 mM, the concentration of the labile Zn pool is less than 20 pM (Maret 2017). In contrast, the size of the exchangeable Co pool in host cells has not been quantified, leaving the extent to which Co is bioavailable to bacteria unresolved. However, several transcriptomic studies have shown that the *PA2911-PA2914* and *PA4063-PA4066* operons are highly expressed in bacteria colonizing human tissues. Since the available experimental data indicate that these operons encode import and export systems selective for the PCH-Co complex, we hypothesize that they play an active role in nutritional immunity and that sufficient Co is available in host tissues to mitigate the effects of Zn deficiency (Secli et al. 2024).

To investigate the role of Co in the response to Zn-starvation in vivo, we employed *G. mellonella* as a model host, as it mimics the nutritional immunity response of vertebrates by restricting Zn availability to invading pathogens (Michetti et al. 2025). Disruption of the outer membrane PCH-Co receptor PA2911 significantly compromises *P. aeruginosa* fitness in this model organism. This is consistent with the previously reported induction of the *PA2911* transcript level in bacteria colonizing larvae (Michetti et al. 2025). Since PA2911-PA2914 is an entry gate for Co-bound PCH, not Zn-bound PCH (Secli et al. 2024), this result suggests that *G. mellonella* can provide a bioavailable Co pool that *P. aeruginosa* can exploit to compensate for Zn starvation during infection. In contrast, inactivation of the PCH-Co exporter PA4065 did not affect bacterial colonization of *G. mellonella* larvae. This result differs from earlier findings showing that disruption of PA4063, a component of the PCH-Co export system (Fiorillo et al. 2021; Secli et al. 2024), markedly reduces *P. aeruginosa* proliferation in airway mucus, indicating that the absence of a functional PCH-Co export system negatively impacts the pathogen fitness (Gi et al. 2015). One possible explanation is that Co concentrations in the larval haemolymph are inherently low and insufficient to impose toxicity, even without the PCH-Co exporter. Collectively, our results suggest that Co can partially compensate for Zn deficiency by substituting Zn in key enzymes and supporting virulence factors like proteases and pyocyanin production. However, its toxicity at higher concentrations or in the presence of a sufficient amount of Zn underscores the need for a tightly regulated metal balance. Understanding these dynamics could provide valuable insights into microbial metal homeostasis and its impact on infection strategies.

## Supplementary Information

Below is the link to the electronic supplementary material.

Supplementary file1 (PDF 757 KB)

## Data Availability

No datasets were generated or analysed during the current study.
