# Supplementary material for: Cobalt mitigates zinc-starvation effects in Pseudomonas aeruginosa
Source: Biometals. 2025 Nov 27;39(2):587–606. doi: 10.1007/s10534-025-00769-4 (PMC13083405; doi:10.1007/s10534-025-00769-4)
Supplement: Supplementary file 1 — Supplementary file1 (PDF 757 KB) [file 10534_2025_769_MOESM1_ESM.pdf]

Figure S1

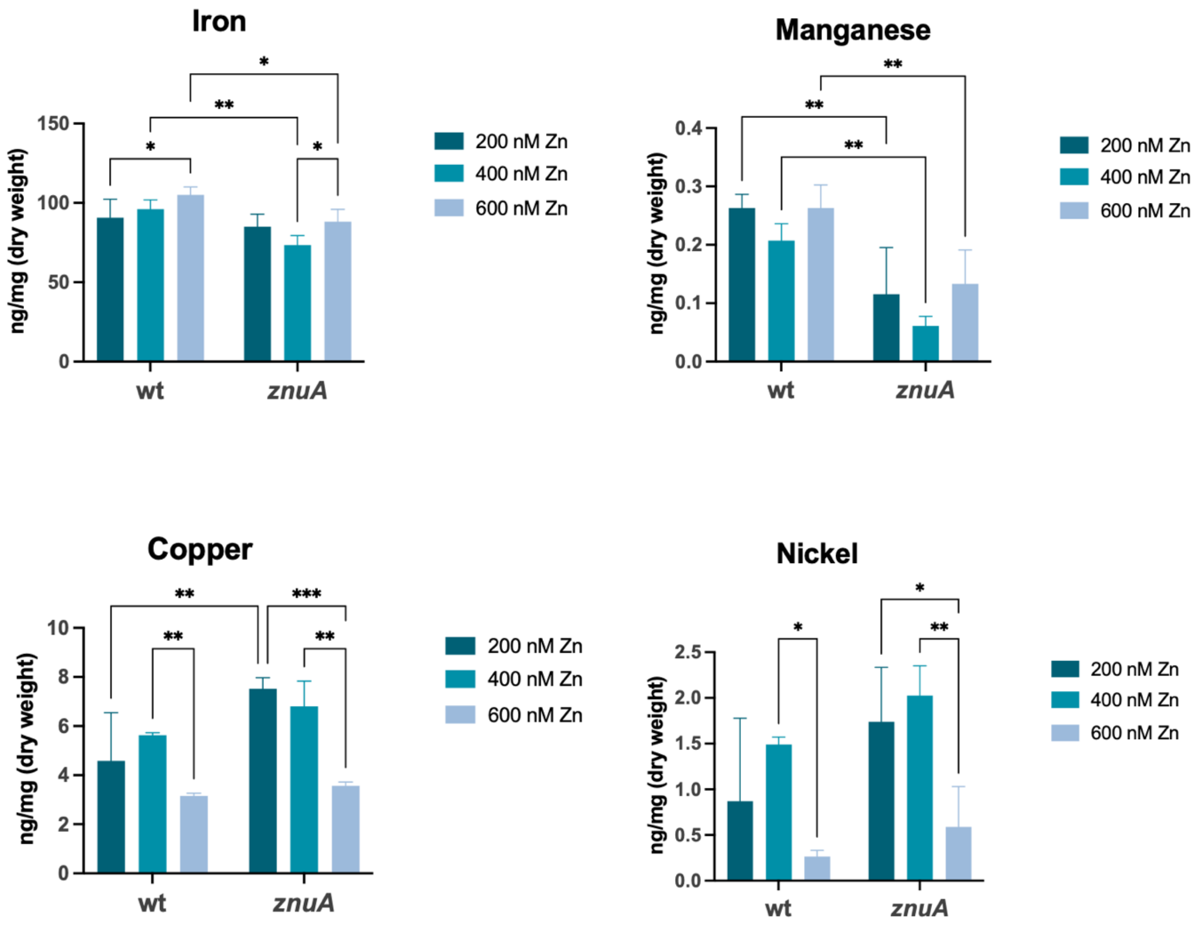

Intracellular metal content in *P. aeruginosa* grown in E-VBMM supplemented with trace metals and increasing $ZnSO_4$ concentrations. Growth conditions are the same as indicated for Fig. 1. Bars are the mean values of three biological replicates ± SD. Statistical analyses were performed using two-way ANOVA and Tukey's multiple comparisons test. Asterisks indicate statistically significant differences between wild-type and *znuA* strains (*$p < 0.05$; **$p < 0.01$; ***$p < 0.001$). The increase in intracellular Cu and Ni levels in the *znuA* mutant strain, which is suppressed under conditions of high Zn availability, is likely explained by the activation of non-selective metal uptake pathways as a compensatory response to Zn deficiency, as previously suggested (Mastropasqua *et al.*, 2017). The decrease in Mn content in the znuA mutant, previously reported and discussed in an earlier work (D'Orazio *et al.*, *Metallomics*, 2015), could be explained by the inhibition of Mn importers by metals such as Cu, Ni or Zn

Figure S2

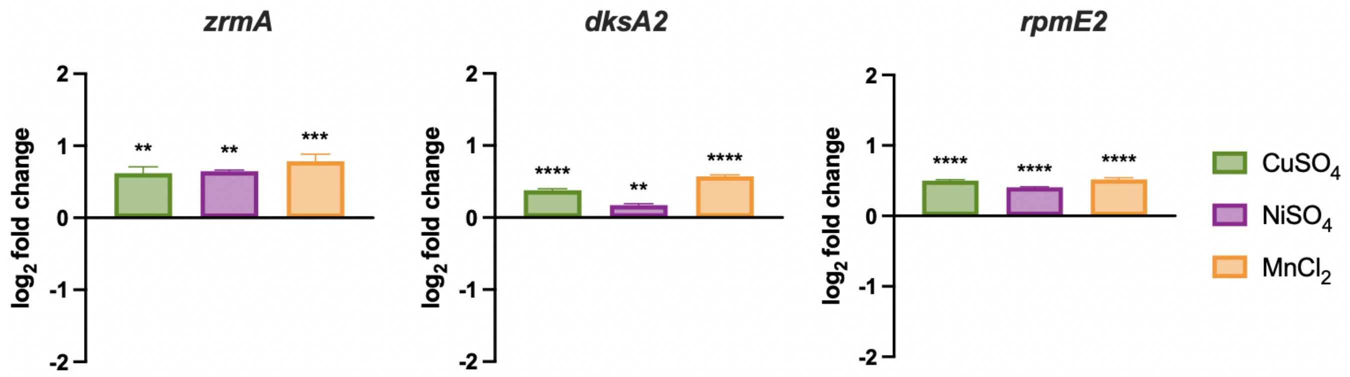

mRNA expression levels of Zur-regulated genes related to metal availability. qRT-PCR on Zur-regulated genes from *P. aeruginosa* PA14 wild-type grown in VBMM supplemented or not with 2 µM metals as indicated in the legend. Data are mean values ± S.D. of triplicates and expressed as Relative fold expression ($log_2\Delta\Delta Ct$) compared to the gene expression in VBMM without metals (control). Statistical analyses were performed using one-way ANOVA and Sidak's multiple comparison test. Asterisks indicate statistically significant differences between each sample and the control (**$p<0.01$; ***$p<0.001$; ****$p < 0.0001$).

Figure S3

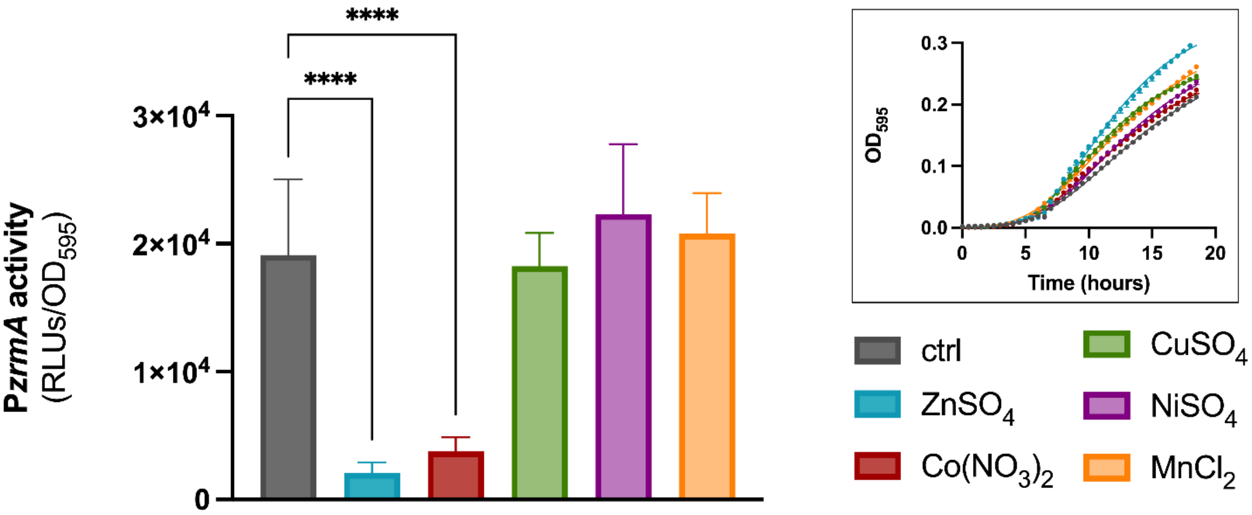

Transcriptional activity of the *zrmA* promoter related to metal availability. Promoter activity was evaluated in PA14 carrying the P*zrmA*-lux reporter plasmid, as previously described (Michetti et al. 2025). The strain was grown in E-VBMM (ctrl) supplemented with metals (7µM) as indicated in the legend. Bacterial growth (OD_{595}, shown in the inset) and luminescence (Relative Luminescence Units, RLUs) were recorded by a Sunrise microplate reader (Tecan). Promoter activity was calculated by the RLUs/ OD_{595} ratio after 18 hours and reported in the graph. The bars represent the average values of two biological replicates, each one tested in triplicate. Statistical analyses were performed using One-way ANOVA, comparing each treatment with the control value (grey bar). ****$p < 0.0001$.

Figure S4

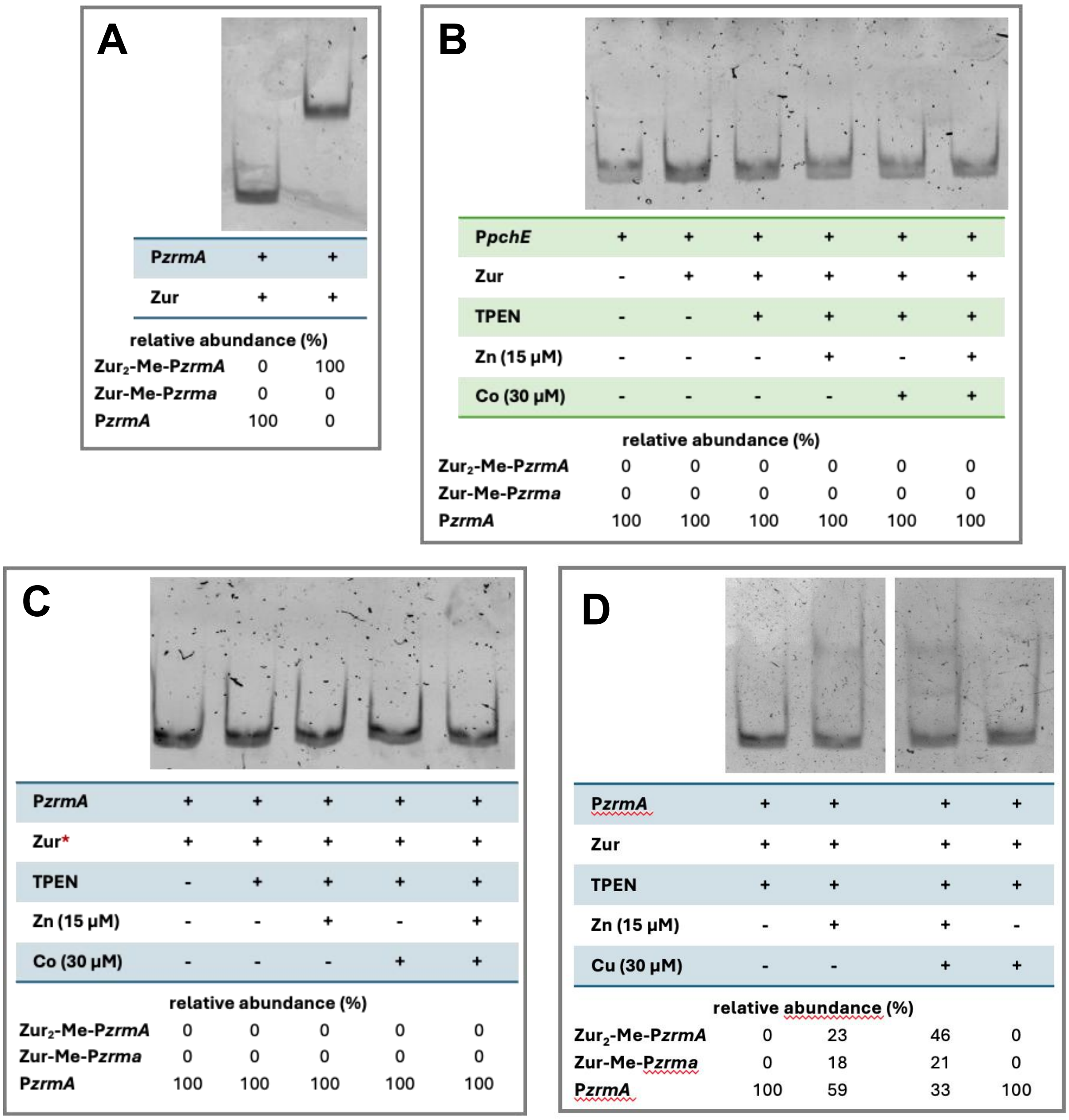

Electrophoretic mobility shift assays (EMSAs) of Zur and DNA. DNA fragments (30 ng) were incubated with purified Zur protein (1 µM), in the presence or absence of the metal chelator TPEN (30 µM) and the indicated metal ($ZnSO_4$, $CoCl_2$, $CuSO_4$). **(A)** Binding of Zur to the *zrmA* promoter in the absence of TPEN and metals. **(B)** Negative DNA control using the Zur-unspecific *pchE* promoter. **(C)** Negative protein control with heat-denatured Zur (Zur*). **(D)** Effect of Cu on Zur–P*zrmA* complex formation in the presence of TPEN and/or Zn. Relative band abundance was quantified using ImageJ and is indicated below each gel as the percentage of total DNA in each lane. All reactions were resolved on 7.5% non-denaturing polyacrylamide gels, stained with ethidium bromide, and visualized under UV light.

Figure S5

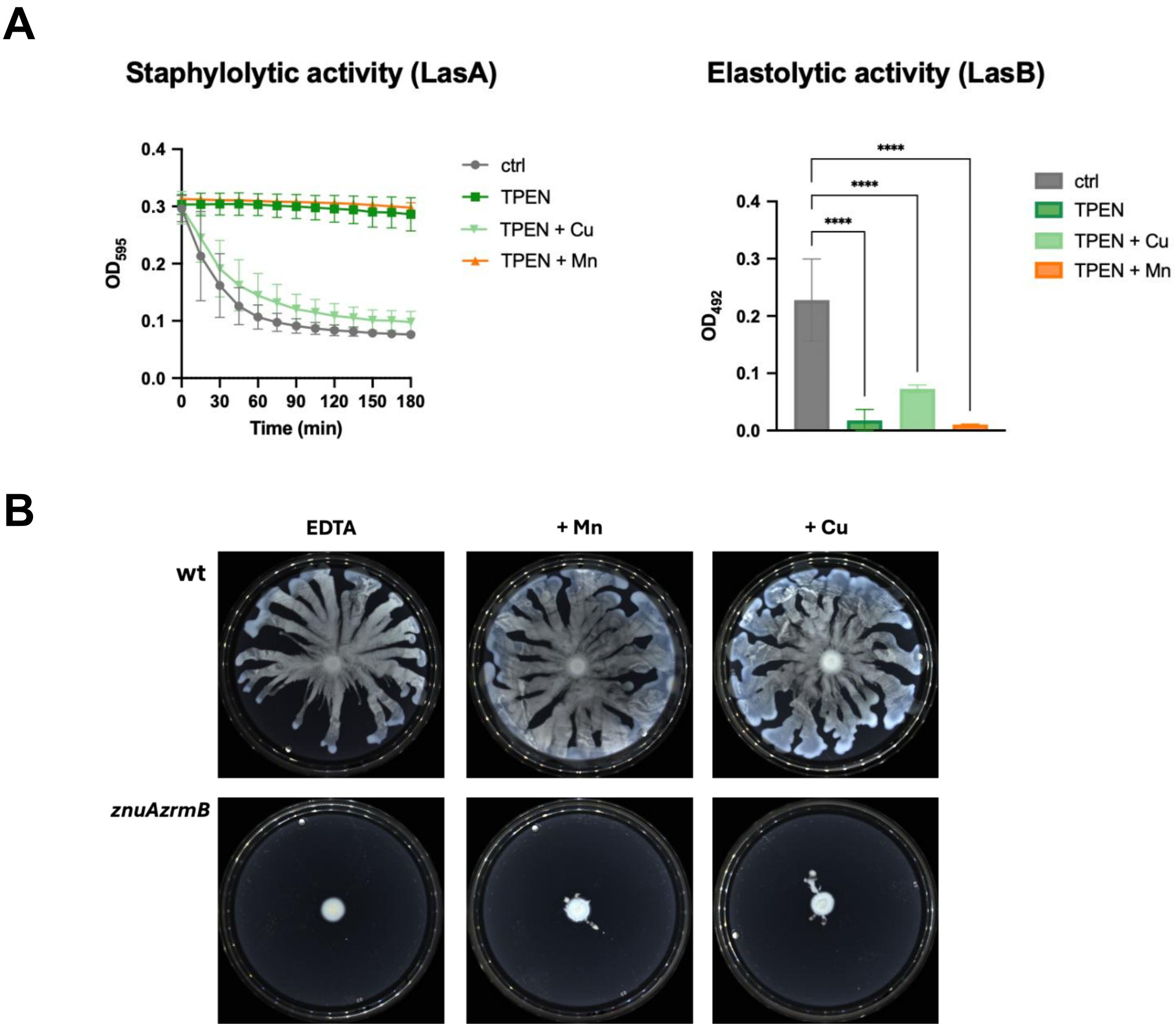

Effect of metals on protease activity and swarming motility of *P. aeruginosa*. (A) Staphylolytic and elastolytic activities were assessed in the supernatant collected from PA14 wild-type grown overnight in ASM. The supernatants were left untreated (ctrl) or treated with 50 µM TPEN, with or without the addition of 500 µM $MnCl_2$ or $CuSO_4$. Data represent mean values from three independent colonies ± S.D. Statistical analysis was conducted using one-way ANOVA and Bonferroni's multiple comparisons test. Asterisks denote statistically significant differences (****$p < 0.0001$). **(B)** Images of swarming plates of PA14 wild-type and *znuAzrmB* mutant strain, containing 10 µM EDTA (ctrl) supplemented with 10 µM $MnCl_2$ or $CuSO_4$. The experiment was repeated three times, with similar results.
